# 44 Predictors of physical activity level among community-dwelling older adults in a suburban Nigerian population

**DOI:** 10.1093/eurpub/ckae114.101

**Published:** 2024-09-26

**Authors:** Fatai Maruf, Christopher Akosile, Yvonne Ihegihu, Olufunke Ajiboye

**Affiliations:** Nnamdi Azikiwe University, Awka, Nigeria; Chrisland University, Abeokuta, Nigeria; Nnamdi Azikiwe University, Awka, Nigeria; Nnamdi Azikiwe University, Awka, Nigeria; Chrisland University, Abeokuta, Nigeria

## Abstract

**Purpose:**

This study explored and determined predictors of PA level among a population of Nigerian community-dwelling older adults

**Methods:**

This cross-sectional study involved 386 community-dwelling older adults (247 females and 139 males) who were recruited at the outreach primary healthcare centre of a teaching hospital. Socio-demographics, Health-related quality of life (HRQoL), fall incidence, fear of falling (FoF), balance, depression, cardiorespiratory fitness (CRF) level, frequency of social visit, number of chronic diseases, number of informal carers, PA level and activities of daily living of participants were assessed using standardised procedures. Multivariate Binary Logistic Regression, using forward likelihood ratio and simple contrast, was used to determine predictors of PA level.

**Results:**

there were lower odds for low PA level among older adults who had ≤2 chronic diseases [odd ratio (OR) 0.17, 95% confidence interval (CI) 0.09-0.32], had low FoF [OR: 0.19, 95% CI 0.05-0.72], had high CRF level [OR 0.12, 95% CI 0.06-0.22], engaged in > 1 social visit per week, [OR: 0.37, 95% CI 0.19-0.70], and had at least secondary education [OR:0.43, 95% 0.19-0.97]. However, those who reported high mental component of HRQoL [OR: 2.98, 95% CI 1.41-6.29] had a higher odd for low PA level.

**Conclusions:**

These findings confirm that having no or fewer diseased conditions, not having FoF, having enough capacity to move around, and having higher educational attainment are predictive of having higher PA level. However, it is counter-intuitive to find that those with higher mental component score of HRQoL are likely to be less physically active. This finding may be explained in light of the possibility that those who are economically advantaged may tend to have to move around less, especially to look for means of livelihood in the rural African context, leading to them being less physically active and yet reporting high mental component of quality of life. Thus, policy to address low PA behavior should be targeted at these groups of individuals in order to rescue them from the tendencies to live sedentary lives.

**Support/Funding source:**

None.

